# Associations Between Suboptimal Social Determinants of Health and Diabetes Distress in Low-Income Patients on Medicaid

**DOI:** 10.1007/s11606-025-09367-z

**Published:** 2025-03-03

**Authors:** Emily H. Williams, Lucia D. Juarez, Caroline A. Presley, April Agne, Andrea L. Cherrington, Carrie R. Howell

**Affiliations:** 1 Tinsley Harrison Internal Medicine Residency Training Program, Department of Medicine, School of Medicine, University of Alabama at Birmingham, 1808 7th Avenue South, Birmingham, AL 35233 USA; 2 Division of Preventive Medicine, Department of Medicine, School of Medicine, University of Alabama at Birmingham, 1717 11Th Avenue South, Birmingham, AL 35205 USA

**Keywords:** social determinants of health, diabetes, diabetes distress, low SES

## Abstract

**Aims:**

To determine associations between suboptimal social determinants of health (SDoH) and diabetes distress in adults with diabetes on Medicaid.

**Methods:**

We surveyed adults with type 2 diabetes covered by Alabama Medicaid. Diabetes distress was assessed using the Diabetes Distress Scale. Suboptimal SDoH included food or housing insecurity; having < high school degree; being unemployed; and household income < $10,000/year. Unadjusted associations between individual SDoH and diabetes distress were examined using logistic regression. We also examined the association between the number of suboptimal SDoH and distress. Multivariable models controlled for age, sex, race, marital status, rurality, diabetes duration, social support, and insulin use.

**Results:**

In total, 433 patients participated (mean age, 50 years (SD 10.4); 80% female; 62% Black). Roughly 32% reported food insecurity, participants experienced a mean of 2 (SD, 0.9; range 0–5) suboptimal SDoH. There was increased odds of diabetes distress in participants who reported food insecurity (OR, 2.2; 95% CI, 1.36–3.65 and OR, 2.35; 95% CI, 1.40–3.93). For each additional suboptimal SDoH a patient experienced, they had increased odds of experiencing diabetes distress (OR, 1.50; CI, 1.15–2.01).

**Conclusions:**

Participants with diabetes who reported food insecurity or experienced a higher number of suboptimal social determinants of health had an increased likelihood of experiencing diabetes distress.

## INTRODUCTION

More than 37 million people in the USA have diabetes and up to 7 million are undiagnosed.^1^ Racial and ethnic minority groups and low-income adults have higher prevalence and incidence of diabetes, along with higher rates of associated complications.^2^ These groups are also more likely to receive suboptimal healthcare for both prevention and treatment of diabetes complications.^2^ Further, the prevalence of diabetes is higher in certain geographic locations in the USA, especially many southeastern states, where racial and socioeconomic disparities are evident.^3^ While disparities are present throughout all regions, previous work has shown that southern states have higher rates of unemployment and generally lower income rates, education levels, and healthcare coverage than non-southern states.^4^

Social determinants of health (SDoH) are defined as the conditions in which individuals live and work and it has been estimated that 45–60% of health disparities are attributable to social determinants.^2,3^ SDoH play a critical role in diabetes-related disparities; factors such as socioeconomic status, physical environment, food environment, healthcare, and social support have all been shown to influence diabetes prevalence and management.^5^ Socioeconomic status (SES) is a SDoH that consistently predicts disease onset and progression for diabetes and many other diseases.^6,7^ Type 2 diabetes prevalence is higher in populations with lower incomes, lower education levels, more single parent households, and crowded housing.^7^ Certain adverse SDoH such as housing instability and food insecurity in people with diabetes have been shown to predict higher outpatient care utilization and poor glycemic control.^8,9^ While race itself is a social construct not considered a SDoH, it is important to acknowledge the history of systemic and institutionalized racism as a SDoH that can negatively impact physical and mental health.^10^

Diabetes distress is defined as the emotional burden related to living with diabetes.^11^ It has been associated with poorer adherence to recommended diabetes treatments and worse glycemic control.^11–14^ Furthermore, in participants with diabetes, the presence of perceived stress, with or without additional depressive symptoms, has been associated with increased risk of acute coronary disease and cardiovascular death.^15^ Interventions such as nurse led mindfulness and stress reduction have been shown to reduce diabetes distress as well as diabetes self-management and self-efficacy.^16,17^ Similarly, the addition of mobile-enhanced peer support interventions to community-based diabetes self-management education (DSME) has been shown to reduce diabetes distress compared to DSME alone.^17^ Additionally, social support has been identified as a mediator of health outcomes, specifically in diabetes.^18^ Previous work has shown that social support influences improved self-care behaviors which in then affects glycemic control.^19,20^ Furthermore, it has been found that elevated diabetes distress is associated with lack of perceived social support.^21^ It is known that intervening in ways that target diabetes distress improves outcomes; however, it is not well understood the way that social support interacts with both diabetes distress and SDoH.^16,17^

Although it has been established that having suboptimal SDoH is associated with worse diabetes outcomes, little research has examined associations between SDoH and diabetes distress. Understanding the relationship between SDoH and diabetes distress may facilitate the identification of individuals who are at particularly high risk, as well as provide intervention targets that may aide in decreasing diabetes distress such as community health workers or other social interventions.^5^ Furthermore, gaining a better understanding of the way that social support may mitigate this relationship may assist in interventions to decrease diabetes distress and therefore improve diabetes outcomes. To address this gap, we sought to evaluate the associations of SDOH and diabetes distress in low-income individuals with diabetes covered by Alabama Medicaid.

## METHODS

### Study Population and Setting

This cross-sectional study consisted of adults 19–64 years of age with type 2 diabetes covered by Alabama Medicaid within the Alabama Care Study Plan. The Alabama Care Plan Study included adults with type 1 or type 2 diabetes that were covered by Alabama Medicaid. Data was gathered from Medicaid enrollment and claims data files to identify potential participants who met the inclusion criteria for the study. For this study, only adults with type 2 diabetes were eligible. The diagnosis of diabetes was ascertained based on the presence of one inpatient or two outpatient International Classification of Diseases (ICD) codes in the preceding 2 years. Participants were excluded if they were physically or mentally incapable of completing the survey, or if they were non-English speaking. These participants were contacted by letter with information on the study and options to decline participation. Interested participants were then contacted by study interviewers to schedule a time to complete the study survey by telephone. All procedures were in accordance with the ethical standards of the International Review Board.

### Diabetes Distress

The primary outcome of the study was perceived diabetes distress measured using the Diabetes Distress Scale (DDS).^22^ This scale consists of 17 items that assess a patient’s diabetes distress over the past month. There are four subscales in the DDS that assess emotional burden, physician-related distress, regimen-related distress, and diabetes-related interpersonal distress. Participants answered on a 6-point scale from 1 (not a problem) to 6 (a very serious problem). A mean score was calculated ranging from 1 to 6 with a DDS score of < 2 indicating low diabetes distress, 2 to < 3 moderate diabetes distress, and ≥ 3 severe diabetes distress.^11^ In our study, participants with scores correlating to moderate or severe diabetes distress were categorized as distressed.

### Social Determinants of Health

Survey information was also used to categorize participants based on whether or not they had suboptimal SDoH. Suboptimal SDoH included (1) experiencing food insecurity, (2) experiencing housing insecurity, (3) making less than $10,000 a year, (4) being unemployed, and (5) having less than a high school degree. Food insecurity was evaluated based on responses to the questions “within the past 12 months we worried whether our food would run out before we got money to buy more” and “within the past 12 months the food we bought just didn’t last and we didn’t have money to get more.” Participants were classified as having food insecurity if they responded yes to one or both questions. To assess housing insecurity, participants were asked “in the past 2 months, have you been living in stable housing that you own, rent, or stay in as part of a household?” and “Are you worried or concerned that in the next 2 months you may NOT have stable housing that you own, rent, or stay in as part of a household?” Participants were classified as having housing insecurity if they reported having unstable housing or reported concern that they may lose stable housing. Information on total household income (wages, salaries, Social Security, retirement, and family assistance) was collected and categorized as < $10,000/year versus > $10,000/year. To assess employment, participants were asked “which of the following best describes your current main daily activities and/or responsibilities.” Those who reported being unemployed, laid off, looking for work, disabled, or unable to work for health reasons were classified as unemployed for our analysis. Finally, to assess education, our survey asked for the highest grade or year of school completed as well as the highest degree earned. Based on responses, participants were categorized as having less than a high school degree, obtaining a high school degree, or obtaining greater than a high school degree.

### Social Support

Participants perceived diabetes-specific level of social support was assessed using the question “How much support do you get for dealing with your diabetes?”.^23–25^ This question was obtained from previous investigation that looked at social support in patients with diabetes.^23^ Participants responded on a scale of 1 (no support) to 5 (great deal of support). Responses of 1 and 2 were classified as “low level of social support” and 3 or higher as “moderate to high level of social support” as done in a previous investigation.^21^

### Covariates

Age, race, ethnicity, sex, marital status, urban versus rural setting, diabetes severity (insulin use and duration), and social support were included as covariates.^26^ Age, race, ethnicity, marital status, diabetes severity, and social support were self-reported. Race and ethnicity were reported as White, Black or African American, Native Hawaiian or Other Pacific Islander, American Indian or Alaska Native, Other. Participants were also able to report “Don’t know/Not sure.” Race and ethnicity collapsed into one category for the regression models, non-Hispanic White and non-White.

### Statistical Methods

Descriptive statistics were calculated to characterize the study population sample. Bivariate associations between those with diabetes distress (DDS ≥ 2) versus those without diabetes distress (DDS < 2) were evaluated using *t*-tests and chi-square or Fisher’s exact tests as appropriate. Logistic regression was used to test the association of each SDoH with diabetes distress in separate unadjusted models. A count was created for each suboptimal SDoH and a separate model examined the association between the number of total SDoH and the presence of diabetes distress. Based on SDoH that were significantly associated with diabetes distress in unadjusted models, we then constructed separate multivariable logistic regression models examining associations between food insecurity and diabetes distress as well as SDoH burden (count of SDoH) and diabetes distress in separate models. In these models, we adjusted for included demographics (age, sex, race/ethnicity, marital status, urban vs rural), diabetes severity (diabetes duration and insulin use), and social support (presence or absence based on survey response). Results are presented as odds ratios (OR) and 95% confidence intervals (95% CI).

## RESULTS

### Descriptive Results

**Figure 1:**
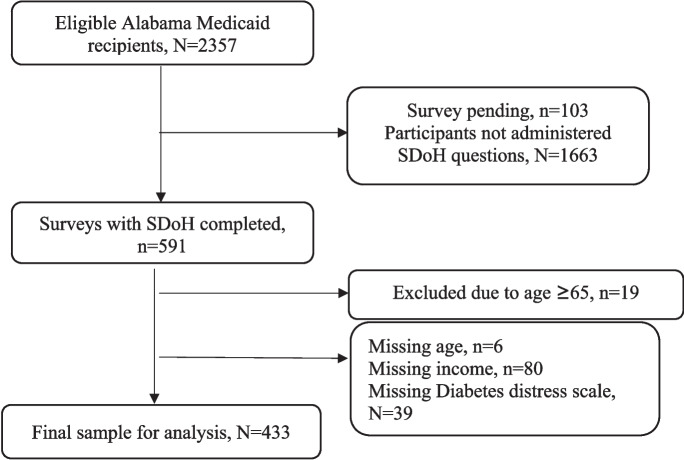
Consort/participant flow.

Figure 1 provides the study consort diagram. In 2019, the AL Care Plan survey was amended to include additional questions about SDoH. Of the 591 surveyed after the questions were added, 19 were excluded due to age > 65 and 6 were excluded due to missing age; 39 were excluded due to missing the outcome of interest (diabetes distress); and 80 participants were excluded because of missing income data. This left a total of 433 individuals for analysis. The average age of participants was 49.1 years (SD 10.29); 80% were female and 62% were Black. Of total participants, 3.2% reported their race as other than White or Black, while 2.1% identified as Hispanic. While HbA1c was not reported in patient surveys, 49.2% of participants reported insulin usage. Participant characteristics are further detailed in Table 1.

**Table 1:** Participant Characteristics of the Study Sample

	Overall	Diabetes distress	No diabetes distress	*p*-value
Age in years, mean (SD)	49.1 (10.29)	46.3 (11.01)	49.7 (10.04)	0.01
Sex (%)				0.27
Male	19.4	15	20.4	
Female	80.6	85	79.6	
Race (%)				0.062
White	33.9	45.0	31.4	
Black	62.6	51.3	65.2	
Other	3.5	3.8	3.4	
Hispanic (%)				0.66
No	98.2	98.8	98.0	
Yes	1.8	1.3	2.0	
**SDoH factors**				
Education level (%)				0.48
< High school	27.9	30	27.5	
High school	43.4	37.5	44.8	
≥ High school	28.6	32.5	27.8	
Income (%)				0.16
< $10,000/year	68.4	75	66.9	
≥ $10,000/year	31.6	25	33.1	
Food insecurity (%)				0.001
Yes	32.3	47.5	28.9	
No	67.7	52.5	71.1	
Housing insecurity (%)				0.49
Yes	1.6	2.5	1.4	
No	98.4	97.5	98.6	
Employment (%)				0.45
Working/studying	86.4	83.8	87.0	
Not working/cannot work	13.6	16.3	13.0	
Rural (%)				0.26
Yes	30.3	25.0	31.4	
No	69.7	75.0	68.6	
Married (%)				0.66
Yes	21.9	23.8	21.5	
No	78.1	76.3	78.5	
Duration of diabetes in years, mean	11.5	12.1	11.3	0.37
Insulin use (%)				0.93
No	50.8	51.3	50.7	
Yes	49.2	48.8	49.3	
No social support (%)				< 0.001
No	86.1	67.5	90.4	
Yes	9.6	32.5	13.9	

### Regression Results

In our sample, 18.5% of participants had moderate-severe diabetes distress; 32.3% reported food insecurity; and participants experienced a mean of 2.2 (SD 0.96) suboptimal social determinants of health. In unadjusted analyses (Table 2), participants who reported food insecurity (OR 2.2, 95% CI 1.36–3.65) or more than one adverse SDOH (OR 1.36, 95% CI 1.04–1.76) had increased odds of experiencing diabetes distress.

**Table 2:** Unadjusted Associations Between SDOH and Diabetes Distress

	Odds ratio	95% confidence interval
SDOH count, per one-unit increment	1.36	1.04–1.76
Education level, *N* (%)		
< High school	1.13	0.66–1.93
≥ High school	Reference	
Employment		
Unemployed	0.77	0.40–1.51
Employed	Reference	
Income < 10 K		
Yes	1.48	0.86–2.58
No	Reference	
Food insecurity, *N* (%)		
Yes	2.23	1.36–3.65
No	Reference	
Housing insecurity, *N* (%)		
Yes	1.79	0.34–9.37
No	Reference	

*SDOH*, social determinants of health

In adjusted models controlling for age, sex, marital status, urban versus rural setting, diabetes severity (insulin use and duration), and social support, participants who reported food insecurity had 2.21-fold higher odds of experiencing moderate-severe diabetes distress (Table 3, CI 1.29–3.76) and 1.5-fold higher odds of experiencing distress for each increase in SDoH (Table 4, CI 1.15–2.02). Notably, in both fully adjusted models, non-White participants had a decreased odds of experiencing diabetes distress, and participants who reported a low level of social support had an increased odds of experiencing diabetes distress even after accounting for SDOH.

**Table 3:** Multivariable Model Examining Associations Between Food Insecurity and Odds of Reporting Diabetes Distress

	Adjusting for demographics only		Adjusting for demographics and disease severity		Adjusting for demographics, disease severity, and social support	
	**OR**	**95% CI**	**OR**	**95% CI**	**OR**	**95% CI**
Food insecurity	**2.33**	**1.40–3.88**	**2.35**	**1.40–3.93**	**2.21**	**1.29–3.76**
Age in years	**0.97**	**0.95–0.99**	**0.97**	**0.94–0.99**	**0.97**	**0.94–0.99**
Male sex	0.70	0.35–1.38	0.72	0.36–1.43	0.59	0.29–1.23
Non-White	**0.51**	**0.30–0.86**	**0.50**	**0.30–0.85**	**0.50**	**0.29–0.85**
Married	0.95	0.52–1.74	0.97	0.53–1.77	0.92	0.49–1.72
Rural zip code of residence	0.67	0.38––1.19	0.66	0.37–1.19	0.62	0.34–1.14
Diabetes duration in years			1.02	0.99–1.05	1.02	0.99–1.05
Insulin use			0.90	0.53–1.54	1.00	0.58–1.74
No social support					**4.69**	**2.51–8.77**

*OR*, odds ratio; *CI*, confidence interval

**Table 4:** Multivariable Model Examining Associations Between the Number of SDOH and Odds of Reporting Diabetes Distress

	Adjusting for demographics only		Adjusting for demographics and disease severity		Adjusting for demographics, disease severity, and social support	
	OR	95% CI	OR	95% CI	OR	95% CI
SDOH count, per one SDoH increase	**1.52**	**1.15–2.01**	**1.52**	**1.15–2.01**	**1.52**	**1.15–2.02**
Age in years	**0.96**	**0.94–0.99**	**0.96**	**0.94–0.98**	**0.96**	**0.93–0.99**
Male sex	0.62	0.31–1.24	0.63	0.31–1.25	0.53	0.25–1.10
Non-White	**0.55**	**0.33–0.92**	**0.54**	**0.32–0.91**	**0.54**	**0.31–0.93**
Married	1.06	0.58–1.93	1.09	0.60–1.98	1.02	0.54–1.91
Rural zip code of residence	0.64	0.36–1.14	0.64	0.36–1.14	0.60	0.33–1.11
Diabetes duration in years			1.02	0.99–1.05	1.02	0.99–1.05
Insulin use			0.80	0.47–1.36	0.90	0.52–1.56
No social support					**5.05**	**2.68–9.52**

*OR*, odds ratio; *CI*, confidence interval

## DISCUSSION

Our study examined the relationship between suboptimal SDoH and diabetes distress in individuals with type 2 diabetes covered by Alabama Medicaid. The participants in our study reported a mean of 2.2 suboptimal social determinants of health, and 18.5% of participants had moderate-severe diabetes distress. Participants who reported food insecurity were 2.2 times more likely to experience elevated diabetes distress than those without food insecurity. For each additional suboptimal SDoH that a participant experienced, there was a 1.5-fold increased likelihood in having moderate-severe diabetes distress. These associations remained even after adjusting for demographics, disease severity, and social support. Notably, non-White participants in our sample exhibited lower odds of diabetes distress after accounting for SDoH and other covariates, while self-reported low social support remained a significant risk factor for diabetes distress. While it is not evident why this association differs based on race, and it is difficult to interpret based on the way race was reported in our sample, it highlights the importance of the relationship between social support and diabetes distress.

The association we observed between food insecurity and diabetes distress is consistent with prior evidence linking food insecurity with a higher prevalence of diabetes distress, depressive symptoms, and lower medication adherence compared to those without food insecurity in participants with poorly controlled diabetes.^27^ High levels of diabetes distress have been associated with worse medication adherence, glycemic control, LDL cholesterol, and diastolic blood pressure^12,28,29^; concomitant food insecurity could contribute to these adverse outcomes directly through impact on diet or indirectly by increasing distress.^27^ Interventions that address food security in those with diabetes have potential to address both a suboptimal SDOH and improved health outcomes. Recent work has shown positive associations with interventions such as home delivery of medically tailored meals for participants with diabetes that has been shown to improve diet quality, bolster food security, and reduce hypoglycemia.^30^ In addition, we found that increases in the number of suboptimal SDOH were associated with an increased risk of diabetes distress. Similarly, in a mixed methods study in 57 participants with uncontrolled glycemia, researchers found that diabetes distress was associated with suboptimal SDOH across multiple domains.^31^

Notably, our study showed a significant association between lack of social support and levels of diabetes distress. This aligns with previous work in the Medicaid population which found an association between poor social support and increased diabetes distress but did not account for SDoH.^21^ Interestingly—and although not categorized a priori as such in our investigation—social support is considered an individual level SDoH. The strong magnitude of effect in our models indicates that lack of social support confers a high risk of diabetes distress even when accounting for food insecurity and other SES indicators which has previously not been well studied. This finding highlights the need to include social support in SDoH screeners when examining diabetes distress and associated clinical outcomes. Moreover, implementing interventions that target social support to ameliorate diabetes distress have potential to improve outcomes. Indeed, such interventions have been shown to play a role in improved self-care practices such as following a healthy diet or committing to an exercise program in both marginalized populations and those with diabetes.^17,23,32^

Examining the relationship between SDoH and diabetes distress highlights the value of screening for social determinants of health, particularly food insecurity and social support, to identify participants who may be at increased risk for developing diabetes distress. By utilizing this type of information in the clinical encounter, healthcare providers can pinpoint social referrals—either to community resources (i.e., food banks), care coordination programs, and make specific adjustments to a medical plan that may help account for SDoH—with the goal to support a patient’s diabetes management and therefore their health outcomes.^33^ Recent work has found that linking individuals to social referrals improved uptake of social services, addressed barriers to healthcare, and helped physician care for participants with chronic conditions.^34,35^ Additionally, patient-tailored motivation and support text message interventions have been associated with greater reductions in HbA1c than usual care alone.^36^ Being able to better identify participants at higher risk for diabetes distress could help guide healthcare providers in employing such interventions.

Our study is not without limitations. Although our study did encompass a large sample population, our sample numbers were limited and focused on participants covered by Medicaid in one state in the southeast USA. Our study did not include participants’ HbA1c, which may make results difficult to interpret and apply to patients with varying control of their diabetes. A study may be able to draw more generalizable conclusions with a larger sample size that encompasses multiple types of health insurance coverage and geographic locations. Additionally, our measurements of SDoH were done based on scales developed from survey responses. The survey questions used varying time frames (e.g., distress measured within 30 days and housing insecurity in last 2 months) which may have impacted the validity in our measurement of SDoH and may not accurately capture the intersectionality of SDoH. Similarly, our measures for social support were subjective and therefore may have left room for participant interpretation about whether that support was coming from social connections rather than medical providers. Lastly, the initial version of the survey did not include SDoH-focused questions, so as these were added later, sample size for this analysis was limited.

Understanding the impact that social determinants of health have on diabetes distress and therefore diabetes management has the potential to improve overall diabetes care. Our study shows that participants who experience food insecurity and who experience a higher number of suboptimal SDoH have an increased likelihood of experiencing diabetes distress. Our findings suggest that identifying participants who have suboptimal social determinants of health may allow healthcare providers and systems to identify participants at risk of elevated diabetes distress that could benefit from targeted interventions.^37^
